
JOURNAL INFORMATION
==============================
NLM Title Abbreviation: ScientificWorldJournal
Journal ID: ScientificWorldJournal
NLM Journal ID: 101131163
Journal ID: TSWJ

The Scientific World Journal

ISSN: 2356-6140
EISSN: 1537-744X
Publisher: Wiley

ARTICLE INFORMATION
==============================
PMCID: PMC6083983
PMID: 30147529
DOI: 10.1100/tsw.2001.168
Article ID: 275809
Article version: 1
Subjects: Short Report

Anticancer Drug-Induced Apoptosis and Cytotoxicity in Prostate Cancer Cells Are Modulated by Organ-Specific Stromal Cell Factors

Lokeshwar Bal L.
Prabhakar Kamalaveni R.
Shang Tie Yan
Mourelatos Zafiria
Li De-Quan
Sylvester Cancer Center and Department of Urology (M-800), University of Miami School of Medicine, Miami, Florida, USA
Publication date: 2001
Electronic publication date: 2001 Oct 31
Volume: 1
First page: 59
Last page: 59
Copyright: Copyright © 2001 Bal L. Lokeshwar et al.
Copyright year: 2001
License: This is an open access article distributed under the Creative Commons Attribution License, which permits unrestricted use, distribution, and reproduction in any medium, provided the original work is properly cited.
License URL: https://creativecommons.org/licenses/by/3.0/

Funding: National Cancer Institute R01-CA 61038 U.S. Army DAMD17-98-18526

==============================
Miami *Nature Biotechnology* Short Reports
*TheScientificWorld* (2001) 1(S3), 59SR
ISSN 1532-2246; DOI 10.1100/tsw.2001.168

### ANTICANCER DRUG-INDUCED APOPTOSIS AND CYTOTOXICITY IN PROSTATE CANCER CELLS ARE MODULATED BY ORGAN-SPECIFIC STROMAL CELL FACTORS

Bal L. Lokeshwar, Kamalaveni R. Prabhakar, Tie Yan Shang, Zafiria Mourelatos, and De-Quan Li

Sylvester Cancer Center and Department of Urology (M-800), University of Miami School of Medicine, Miami, Florida
Blokeshw@med.miami.edu

**INTRODUCTION.** Most anticancer drugs kill tumor cells by inducing apoptotic cell death [1]. Enhancement of this apoptosis-inducing activity or overcoming tumor's resistance to apoptosis induction by anticancer drugs could bring a significant improvement in cancer therapy. Cytokines and tissue-specific factors mediate communication between tumor cells and surrounding stromal cells [2,3]. These agents are believed also to modulate tumor cells' response to anti-cancer drugs. This phenomenon is predominantly acute in metastatic prostate cancer, where most patients with metastatic disease survive less than 3 years. The purpose of this study was to examine the impact of stromal cells (fibroblast and endothelial cells)-induced cytokines on the response of tumor cells to anticancer drugs.

**METHODS.** The consequence of interaction between stromal cells and tumor cells with respect to tumor cells' response to three anti-tumor drugs (COL-3, doxorubicin, and taxol) was investigated in three established prostate cancer (CaP) cell lines (LNCaP, DU 145 and PC-3). Modulation of drug-induced cytotoxicity and apoptosis in CaP cells by purified cytokines and stromal cell-conditioned media (SCM) were investigated. Conditioned media were prepared from primary fibroblasts and microvessel endothelial cells (EC) cultures of human skin, lung and prostate. Conditioned media and co-cultures of rat bone-marrow fibroblasts and mouse 3T3 fibroblasts cultures were also tested. Cytotoxicity was quantified by Thiazolyl blue (MTT) reduction assay or inhibition of [$^{3}$H]-methyl thymidine incorporation. Histone -1 released due to apoptotic activity was measured using the Celldeath-ELISA-Plus kits (Roche, NJ). Levels of cytokines in various treatment groups were quantified using commercial ELISA-kits. Modulation of IL-1$ and IL-6 mRNA in tumor cells by SCM and cytokines were compared using a semi-quantitative RT-PCR [3].

**RESULTS.** SCM derived from primary and established fibroblast cultures of skin, lung, bone marrow and prostate origin were able to reduce or increase drug-induced cytotoxicity in all the three CaP cell lines. Mouse 3T3 fibroblast CM and human skin and prostate-derived SCM *increased* cytotoxicity of anticancer drugs by 20-80%, compared to regular complete culture medium. Cytotoxicity of taxol was least affected compared to COL-3 (a chemically modified non-antimicrobial tetracycline with potent anti-tumor activity [4]. (Cytotoxicity due to doxorubicin was affected moderately by SCM or cytokines. Human lung and bone marrow-derived SCM *reduced* drug-induced cytotoxicity by 1.5 to 3-folds. Cytotoxicity and drug-induced apoptosis was higher in the presence of 3T3 cells, human lung and skin fibroblast cocultures but was unaffected in prostate stromal cells and microvessel endothelial cells. These SCMs were able to induce IL-6 and IL-1$ in tumor cells, that was partially inhibited by neutralizing anti-IL-6 and anti-IL-1$ antibodies. However, the inhibition was modest. Furthermore, IL-6 and Il-1$ levels were elevated in CaP cell conditioned medium when treated with drugs, but was increased further, up to 5-fold, in the presence of SCMs. Expression of cytokine-specific mRNA was significantly elevated in the presence of SCM. In all cases, as revealed by a semi-quantitative RT-PCR, the SCM that sensitized tumor cells to drugs increased IL-6 mRNA in tumor cells (DU145 and PC-3) (Fig. 1). Anti-IL-6 antibody could only partially (#50%) reverse SCM-induced drug sensitivity, indicating other factors (being identified by gene expression array analysis) may also be responsible for modulation of cytotoxicity. Similar activity was observed in the case of IL-1$ expression in the presence of anti-cancer drug (e.g., taxol) and SCM (Fig. 2). A significant increase in cytotoxicity and drug-induced apoptosis with a concomitant increase in IL-6 and IL-1$ mRNA transcripts was observed in CaP cells exposed to drugs in the presence of skin fibroblasts, microvessel endothelial cell conditioned medium (Fig. 2).

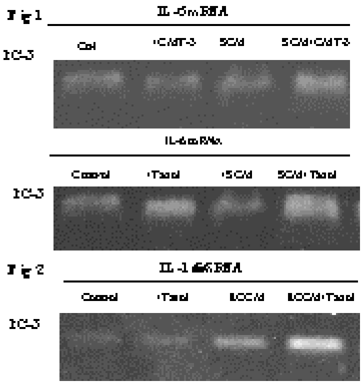

**CONCLUSIONS.** These results indicate that the effect of anticancer drugs can be significantly altered by stromal-derived factors that act by inducing IL-6 and IL-I$ in tumor cells, which may trigger additional pathways of drug-induced cytotoxicity and apoptosis. Further understanding of the mechanism and impact of organ-specific stromal factors should be able to reduce the failure of chemotherapy in metastatic malignancy. [Work supported by PHS (NCI) R01-CA 61038 and DoD/ US Army DAMD17-98-18526 grants.]

**REFERENCES.**

1. Kamesaki, L. (1998) Int. J. Hematol. 68, 29-43
2. Liu, P. et al. (2000) Int. J. Oncol. 16, 599-610
3. Zhu, B.-Q., Block, N.L., and Lokeshwar, B.L. (1999) Ann. N. Y. Acad. Sci. 878, 642-646
4. Lokeshwar, B.L. (1999) Ann. N. Y. Acad. Sci. 878, 271-289
